# Physicians’ Opinion and Practice of Vitamin K Administration at Birth in Romania

**DOI:** 10.3390/healthcare10030552

**Published:** 2022-03-16

**Authors:** Andreea Avasiloaiei, Demetra Gabriela Socolov, Maria Stamatin, Mihaela Moscalu

**Affiliations:** 1 Mother and Child Health Department, “Grigore T. Popa” University of Medicine and Pharmacy, 700115 Iasi, Romania; socolov@hotmail.com (D.G.S.); maria.stamatin@yahoo.com (M.S.); 2 Preventive Medicine and Interdisciplinary Department, “Grigore T. Popa” University of Medicine and Pharmacy, 700115 Iasi, Romania; mihaela.moscalu@umfiasi.ro

**Keywords:** vitamin K, neonatal hemorrhage, vitamin-K-deficiency bleeding, survey, parental refusal, prophylaxis

## Abstract

(1) Background. Vitamin K is recommended worldwide as a standard of care for the prophylaxis of vitamin-K-deficiency bleeding (VKDB). This is also a standard practice in Romania, but due to the rising number of refusals by parents of basic interventions in the neonatal period, we aimed to assess the Romanian neonatologists’ opinions and current practice regarding vitamin K administration at birth. (2) Methods. We designed and conducted an electronic survey addressed to 110 physicians working in Romanian hospitals. (3) Results. Half of respondents are accustomed to receiving refusals for vitamin K administration once or twice a year. When parents refuse vitamin K administration, they usually refuse other neonatal interventions, according to 90.9% of the responding physicians, and this situation has occurred more frequently during the last two years. The number of refusals and especially their increase are more frequent in level III hospitals (*p* = 0.0304, *p* = 0.0036, respectively). Only 22.7% of the physicians responded that they would recommend an oral preparation of vitamin K in the absence of intramuscular prophylaxis. (4) Conclusion. Efforts should be made to address parents’ concerns and to have available alternatives to the intramuscular administration of vitamin K.

## 1. Introduction

Vitamin K is administered worldwide in hospitalized deliveries to prevent the onset of vitamin-K-deficiency bleeding (VKDB). Although, for the most part, VKDB can manifest with less significant cutaneous or gastrointestinal hemorrhaging [1], it can also potentially be a debilitating condition, especially in its late onset [2,3]. Prophylaxis is therefore sorely desired. Vitamin K administration via intramuscular injection is highly efficient in preventing VKDB, but the administration route is sometimes considered an issue, especially due to the potential incidents of muscle damage or pain caused to the neonates [4].

Vitamin K has been used for VKDB prophylaxis since the 1940s [5] and in 1961 the American Academy of Pediatrics issued a statement paper [6], recommending vitamin K administration at birth as a standard of care. In terms of healthcare policy, VKDB prophylaxis is still considered extremely effective due to its high efficiency and availability [7].

Intramuscular administration is the preferred route, due to optimal storage and slow release [8], but concerns arose in the 1990s, starting with studies by Golding et al. [9,10], which linked intramuscular vitamin K to childhood cancers. Although these findings were subsequently refuted [11,12,13], some case-control studies performed later were unable to deny this assumption, because of ethical issues and multiple confounding factors.

However, due to these concerns, as well as the aforementioned adverse effects of intramuscular administration, alternative ways of administering vitamin K were researched, including oral preparations. These are now used as part of the standard regimen of administration in infants in many countries of the world [14]. Although the oral route is by far more comfortable, it is constantly deemed less efficacious in terms of the incidence of late VKDB, when compared to the intramuscular route [15].

In Romania, the regimen for vitamin K administration is via intramuscular injection for healthy newborns as a single dose immediately after birth or the intravenous route for sick neonates as repeated doses during the first week of life. Oral preparations for neonatal use are not widely available.

We sought to ask neonatologists working in Romanian hospitals what is their opinion and what are the challenges they face when performing VKDB prophylaxis in their daily practice. Moreover, we aimed to find out whether the level of care in the hospitals our respondents work influences their practice regarding vitamin K prophylaxis.

## 2. Materials and Methods

### 2.1. Study Population

Based on previous literature review, we conceived a questionnaire made up of 10 items, which we addressed via the Internet as part of an online CME event to a group of 157 physicians, members of the Romanian Association of Neonatology, all of which activate as pediatricians or neonatologists in Romanian hospitals. The questionnaire was open for answers for 12 days (23 July–4 August 2020) and we received a total of 110 anonymous answers.

### 2.2. Statistical Analysis

Statistical data analysis was performed in SPSS v.25 (IMB Corporation, Armonk, NY, USA). The variables analyzed were qualitative, described as absolute frequencies (n) and relative frequencies (%). For performing the comparisons between three groups, the Chi-square test was applied. A value lower than 0.05 for *p* indicated a statistically significant difference.

## 3. Results

The ten items of the survey, along with the answers we received for each of them are listed in Table 1. The response rate to our questionnaire was 70%. Out of the 110 respondents, 50.9% work in a Level III hospital, 39.1% in a Level II facility, and 10% in a Level I hospital. Moreover, 93.6% stated that their practice is in a public hospital and 6.4% in a private facility.

Informed consent specifically regarding the administration of vitamin K is requested by 66.4% of the respondents and 50.9% of them are accustomed to receiving refusal for vitamin K administration once or twice a year. When faced with refusals, 70% talk to parents in greater detail about hemorrhagic disease signs and symptoms and 81.8% of the respondents answered that they had cases in which parents that refused subsequently changed their mind. According to Romanian physicians, the main reasons for parents’ refusal are: perceiving vitamin K administration as a vaccine (41.8%), their lack of knowledge regarding the usefulness of vitamin K in preventing neonatal hemorrhagic disease (36.3%), and their perception for the lack of necessity for vitamin K supplementation (35.4%). When facing refusals for intramuscular administration, most of the doctors (69.1%) refer the parents to their family physician for supplementary surveillance.

When parents refuse vitamin K administration, they usually refuse other interventions in the neonatal period, according to 90.9% of the responding physicians, and this situation has occurred, in the opinion of 83.4%, more frequently during the last two years.

The correlations between the physicians answers and the level of care of their activity are shown in Table 2. The correlation analysis showed that the number of refusals is associated to the level of care of the hospital the physicians work in—rarer in level I and II facilities (reported as ”once or twice a year” in 72.7% and 55.8%, respectively) and more frequent in level III hospitals, as declared by 51.8% of level III respondents. Moreover, 94.6% of clinicians in level III hospitals reported this situation as being more frequent in the last two years, compared to only 54.5% in level I hospitals and 76.7% in level II facilities, which was deemed statistically significant (*p* = 0.0036).

All the physicians from level I hospitals claimed that parents who refuse vitamin K administration to their infants usually deny other important neonatal interventions, such as vaccines or blood-spot neonatal screening, compared to 83.7% in level II facilities (*p* = 0.0394).

## 4. Discussion

Vitamin K has a long history of being administered via the intramuscular route in order to prevent vitamin-K-deficiency bleeding in neonates. In fact, this route is currently recommended as the most effective by numerous national and international societies [16,17,18,19].

In a wide majority of cases, vitamin-K-deficiency bleeding, which can cause, especially in its late form, debilitating intracranial hemorrhages [20,21], has been virtually eliminated in Romania. However, the increasing number of refusals from parents can lead to a resurgence in VKDB, with potentially fatal consequences for the newly born infants. We wanted to conduct a nation-wide survey in order to confirm this increase and to find out the reasons why parents consider vitamin K administration a risky or unnecessary practice.

The first item of the survey (Q1) aimed to assess whether it is current practice for Romanian physicians to ask for the informed consent of the parents (mostly mothers) specifically for the administration of vitamin K. Our respondents answered affirmatively in 66.4%. The ones who responded negatively probably aim for a much more generalized informed consent, for ”procedures performed at birth”, which also include vaccination for hepatitis B. Until recently, the general consensus in Romania was that unless specifically refused, vitamin K was automatically given. The recent eagerness of physicians to ask for parents’ permission is at least partly due to their trying to avoid the rising number of malpractice accusations.

The specific consent for vitamin K administration is more often requested (*p* = 0.0154) in level I and II hospitals (72.7% and 74.4% respectively), compared to level III hospitals (58.9%), probably due to the already busy workload in the latter, in which neonatologists deal with more complex cases.

More than half of respondents affirmed on Q2 that the situation where parents refuse administration of vitamin K occurs once or twice a year and only in 2 cases (1.8%) when physicians said they stumble upon this situation every week. However, on Q8, 83.6% agreed that refusal has currently increased in frequency.

There is currently no data in our country regarding the actual incidence for refusal of vitamin K prophylaxis, but the increasing trend which was observed by our respondents is nonetheless alarming and confirmed by research [22]. One study from New Zealand over the course of four years reports a constant incidence of refusals [23], with an incidence of 1.7% newborns for whom vitamin K intramuscular administration was declined, but with a rising trend in oral uptake.

As stated in previous studies, VKDB refusal by parents has profound implications on healthcare, as these parents are more prone to refuse other medical procedures in the neonatal ward and beyond [24,25]. This also was deemed true by this survey, where respondents’ opinion in 90.9% of cases (Q9) was that when parents refuse vitamin K administration, they also refuse other procedures during admission, such as vaccinations (hepatitis B and BCG) or dried blood spot screening for congenital metabolic disorders.

Refusal is met with caution by 70% of our respondents, according to their answers to Q3, as they try to provide parents with more information about VKDB, the signs, symptoms, and severity of the disease and give them time to change their minds, which sometimes happens, according to 81.8% of the responses on Q8. This situation is particularly true in level III maternities (*p* = 0.0264), where 80.4% further try to convince parents of the benefits of vitamin K administration, probably due to the academic character of most of level III hospitals. As a direct result, 92.9% from level III respondents claim to have determined parents to accept vitamin K prophylaxis. On the other hand, 24.5% simply acknowledge the refusal and respect the family’s choices. The danger arising from this attitude is linked to the lack of knowledge most of the parents exhibit toward the potentially life-threatening course of VKDB [22].

The fourth item on the questionnaire (Q4) is a multiple-choice question and it explores the potential reasons for which parents might refuse administration of vitamin K. According to Romanian doctors, the reasons for refusal are: perceiving vitamin K administration as a vaccine (41.8%), their lack of knowledge regarding the usefulness of vitamin K in preventing neonatal hemorrhagic disease (36.3%), and their perception for the lack of necessity for vitamin K supplementation (35.4%), concerns regarding preservatives used in the intramuscular injection (11.8%), concerns regarding the long-term effects of pain inflicted by intramuscular injection (4.5%), concerns regarding the association of intramuscular administration to childhood neoplasia (1.8%). None of the respondents reported the dose of vitamin K as being a matter of concern for the parents. About 5.5% of the physicians related other reasons for refusal on Q5, which was an open-answer question: mothers supplementing their prenatal diets with foods rich in vitamin K, concerns about mother’s thrombophilia and its influence on the infant’s bleeding, misunderstandings about vitamin K being a hormone or leading to jaundice, religious beliefs. One answer referred to the influence of other people, family or close friends, on the decision regarding the administration of vitamin K. Interestingly, most of the answers on Q5 came from physicians working in private hospitals.

In a study investigating the reasons for refusal of administration of vitamin K to neonates [26], the authors report concerns over synthetic or toxic ingredients, excessive dose, the belief that it was “unnatural,” and also potential side effects. Another study reported refusals over lack of understanding the indication for the administration, belief that the injection is unnecessary, concern about pain from the injection, and about harm to the infant caused by preservatives [24].

According to the study by Loyal et al. [27], factors linked to parents’ refusal of vitamin K administration were: exclusive breastfeeding, white race, female gender, gestational age, and mother’s age. The mode of delivery and the type of insurance (public/private) did not influence the acceptance of vitamin K prophylaxis. On the other hand, in a retrospective cohort study performed in New Zealand [23], the ethnicity and the mode of birth was correlated to the uptake of vitamin K prophylaxis, while no other factor played a significant part. In New Zealand, in a study designed to explore in more detail the reasoning of parents who refuse vitamin K prophylaxis [28], the authors were able to cluster those reasons into three main themes: parents’ beliefs and values (philosophy and spirituality) which are immersed in many other aspects of the family’s life, concerns about their child’s welfare (pain and potential side effects), and external influencing factors (family, friends, media and health professionals), all of which we were able to find indirectly, in our research.

On Q7, only 22.7% of the physicians responded that they would recommend an oral preparation of vitamin K in the absence of intramuscular prophylaxis. This was the case regardless of the level of maternity hospital the respondents work and is understandable in our country, due to the relative unavailability of vitamin K1 oral solutions.

Vitamin K can be administered orally and there are multiple regimens available; nonetheless, the oral administration is known to be less effective than intramuscular injection [8] therefore multiple doses are needed throughout the neonatal period if oral administration is used. This can be cumbersome for new parents and act as a potential source for their lack of compliance. Moreover, incomplete oral prophylaxis has been detected in newborns with VKDB receiving the oral regimen [29].

Even if throughout Western Europe this practice is acceptable and recommended by some of the national societies of Neonatology or Pediatrics [14], there are countries which renounced to the policy of oral prophylaxis [30,31,32] after a relapse in the number of cases of late-onset VKDB.

In the Unites States, oral vitamin K is not as widely used and is mostly recommended when parents refuse the intramuscular injection [33], although physicians have mixed feelings and knowledge about the ability of oral intake to actually prevent VKDB. Their situation reflects on a grander scale the one in Romania.

Given the lack of consensus on the mode of VKDB prophylaxis, it is probably safer if national guidelines based on individual circumstances were available and reinforced. Our survey was a first step in Romania to find out the current situation and physicians’ knowledge on the subject.

Our study has several limitations. We addressed in our survey only physicians, as other healthcare professionals (midwives/nurses) are not customarily part of the decision-making process in our country, although their opinion may be valuable for some patients. We based our results only on clinicians kind enough to answer our survey in a limited timeframe, regardless of their experience or location. Moreover, this study has a real memory bias due to it being based on doctors remembering their interactions with parents over a long period of time.

## 5. Conclusions

More than half of Romanian neonatologists are accustomed to refusals for vitamin K administration and only some recommend oral vitamin K as an alternative following discharge. Many practitioners observed a rising tendency for parental refusal during the last two years, due to a variety of reasons. Efforts should be made by the healthcare system to ensure proper information reaches the parents and addresses their expressed questions and concerns. Moreover, the lack of oral vitamin K preparations makes it even harder for any kind of prophylaxis to be available, if needed.

## Figures and Tables

**Table 1:** Descriptive analysis of the answers to the survey.

Item	*n* (%)
**Q1: Do you usually ask for parents’ informed consent specifically for vitamin K administration?**	
Q1.1: Yes	73 (66.4)
Q1.2: No	37 (33.6)
**Q2: Did you ever have parents refuse vitamin K administration to their infants?**	
Q2.1: Never	12 (10.9)
Q2.2: Once or twice a year	56 (50.9)
Q2.3: Once every 3 to 4 months	23 (20.9)
Q2.4: Once a month	5 (4.5)
Q2.5: Two or three times a month	12 (10.9)
Q2.6: Every week	2 (1.8)
**Q3: How do you manage refusals?**	
Q3.1: I simply acknowledge the signed refusal and act accordingly, thus respecting parents’ wishes	27 (24.5)
Q3.2: I talk to the parents in greater detail about the signs and symptoms of hemorrhagic disease	77 (70)
Q3.3: I ask about the reason for refusal	5 (4.5)
Q3.4: I recommend supplementary reading material	1 (0.9)
**Q4: Which are the reasons for the parents’ refusal to vitamin K administration?**	
Q4.1: Their perception for the lack of necessity for vitamin K supplementation	39 (35.4)
Q4.2: Their lack of knowledge regarding the utility of vitamin K in preventing neonatal hemorrhagic disease	40 (36.3)
Q4.3: Concerns regarding preservatives used in the intramuscular injection	13 (11.8)
Q4.4: Concerns regarding the long-term effects of pain inflicted by intramuscular injection	5 (4.5)
Q4.5: Concerns regarding the association of intramuscular administration to childhood neoplasia	2 (1.8)
Q4.6: Perceiving vitamin K administration as vaccine	46 (41.8)
Q4.7: Perceiving the dose of vitamin K as being too large	-
Q4.8: Other reasons	6 (5.5)
** Q5: If you indicated ”Other reasons” on the precedent question, please specify **	
** Q6: Did you have situations when parents initially refuse, but change their minds afterwards? **	
Q6.1: Yes	90 (81.8)
Q6.2: No	20 (18.2)
** Q7: When facing refusal of vitamin K intramuscular administration, do you ever recommend an oral preparation as alternative? **	
Q7.1: Yes	25 (22.7)
Q7.2: No, I don’t believe in the efficiency of oral preparations	9 (8.2)
Q7.3: No, but I am careful to inform the family physician about the lack of prophylaxis at birth	76 (69.1)
** Q8: Do you believe that the situation when parents refuse vitamin K administration is more frequent in the last two years compared to before? **	
Q8.1: Yes	92 (83.6)
Q8.2: No	18 (16.4)
** Q9: When parents refuse vitamin K administration, do they usually also refuse vaccinations or screening for congenital metabolic disorders? **	
Q9.1: Yes, they refuse all interventions, albeit diagnostic or therapeutic	100 (90.9)
Q9.2: No, they specifically refuse vitamin K administration	10 (9.1)
**Q10: What is the level of care and type of the hospital in which you work?**	
Q10.1: Level I	11 (10)
Q10.2: Level II	43 (39.1)
Q10.3: Level III	56 (50.9)
Q10.4: Public hospital	103 (93.6)
Q10.5: Private hospital	7 (6.4)

**Table 2:** Correlation of answers to the level of care.

Item	Level of Care			*p*-Value
	Level I (*n* = 11)	Level II (*n* = 43)	Level III (*n* = 56)	
** Q1: **				
Q1.1	8 (72.7)	32 (74.4)	33 (58.9)	** 0.0154 **
Q1.2	3 (27.3)	11 (25.6)	23 (41.1)	** 0.0267 **
** Q2: **				
Q2.1	1 (9.1)	8 (18.6)	3 (5.4)	** 0.0381 **
Q2.2	8 (72.7)	24 (55.8)	24 (42.9)	** 0.0304 **
Q2.3	2 (18.2)	5 (11.6)	16 (28.6)	** 0.0299 **
Q2.4	0 (0)	1 (2.3)	4 (7.1)	** 0.0406 **
Q2.5	0 (0)	4 (9.3)	8 (14.3)	** 0.0282 **
Q2.6	0 (0)	1 (2.3)	1 (1.8)	0.0873
** Q3: **				
Q3.1	4 (36.4)	14 (32.7)	9 (16.1)	** 0.0189 **
Q3.2	7 (63.6)	25 (58.1)	45 (80.4)	** 0.0264 **
Q3.3	0 (0)	3 (6.9)	2 (3.5)	0.0681
Q3.4	0 (0)	1 (2.3)	0 (0)	0.0847
** Q4: **				
Q4.1	3 (27.3)	15 (35.7)	21 (38.2)	0.8971
Q4.2	4 (36.4)	12 (28.6)	24 (43.6)	** 0.0305 **
Q4.3	1 (9.1)	7 (16.7)	5 (9.1)	** 0.0421 **
Q4.4	0 (0)	0 (0)	5 (9.1)	** 0.0125 **
Q4.5	1 (9.1)	1 (2.4)	0 (0)	** 0.0267 **
Q4.6	3 (27.3)	18 (42.9)	25 (45.5)	** 0.0355 **
Q4.7	-	-	-	
Q4.8	0 (0)	5 (11.9)	1 (1.8)	** 0.0194 **
** Q5: **	-	-	-	-
** Q6: **				
Q6.1	9 (81.8)	29 (67.4)	52 (92.9)	** 0.0035 **
Q6.2	2 (18.2)	14 (32.6)	4 (7.1)	** 0.0084 **
** Q7: **				
Q7.1	3 (27.3)	14 (32.6)	8 (14.3)	** 0.0245 **
Q7.2	1 (9.1)	2 (4.7)	6 (10.7)	** 0.0306 **
Q7.3	7 (63.6)	27 (62.7)	42 (75)	0.0974
** Q8: **				
Q8.1	6 (54.5)	33 (76.7)	53 (94.6)	** 0.0036 **
Q8.2	5 (45.5)	10 (23.3)	3 (5.4)	** 0.0021 **
** Q9: **				
Q9.1	11 (100)	36 (83.7)	53 (94.6)	** 0.0394 **
Q9.2	0 (0)	7 (16.3)	3 (5.4)	** 0.0144 **

## Data Availability

Datasets are available upon request from the correspondent author.
